# Protection of the vascular endothelium in experimental situations

**DOI:** 10.2478/v10102-011-0005-y

**Published:** 2011-03

**Authors:** Ružena Sotníková, Jana Nedelčevová, Jana Navarová, Viera Nosáĺová, Katarína Drábiková, Katalin Szöcs, Peter Křenek, Zuzana Kyseĺová, Štefan Bezek, Vladimír Knezl, Ján Dřímal, Zuzana Brosková, Viera Kristová, Ĺudmila Okruhlicová, Iveta Bernátová, Viktor Bauer

**Affiliations:** 1 Institute of Experimental Pharmacology & Toxicology, Slovak Academy of Sciences, Bratislava, Slovakia; 2 Faculty of Pharmacy, Comenius University, Bratislava, Slovak Republic; 3 Faculty of Medicine, Comenius University, Bratislava, Slovak Republic; 4 Institute of Heart Research, Slovak Academy of Sciences, Bratislava, Slovak Republic; 5 Institute of Normal and Pathological Physiology, Slovak Academy of Sciences, Bratislava, Slovak Republic

**Keywords:** diabetes, ischaemia/reperfusion, pyridoindole antioxidans, SMe1EC2

## Abstract

One of the factors proposed as mediators of vascular dysfunction observed in diabetes is the increased generation of reactive oxygen species (ROS). This provides support for the use of antioxidants as early and appropriate pharmacological intervention in the development of late diabetic complications. In streptozotocin (STZ)-induced diabetes in rats we observed endothelial dysfuction manifested by reduced endothelium-dependent response to acetylcholine of the superior mesenteric artery (SMA) and aorta, as well as by increased endothelaemia. Changes in endothelium-dependent relaxation of SMA were induced by injury of the nitric oxide radical (·NO)-signalling pathway since the endothelium-derived hyperpolarising factor (EDHF)-component of relaxation was not impaired by diabetes. The endothelial dysfunction was accompanied by decreased ·NO bioavailabity as a consequence of reduced activity of eNOS rather than its reduced expression. The results obtained using the chemiluminiscence method (CL) argue for increased oxidative stress and increased ROS production. The enzyme NAD(P)H-oxidase problably participates in ROS production in the later phases of diabetes. Oxidative stress was also connected with decreased levels of reduced glutathione (GSH) in the early phase of diabetes. After 10 weeks of diabetes, adaptational mechanisms probably took place because GSH levels were not changed compared to controls. Antioxidant properties of SMe1EC2 found *in vitro* were partly confirmed *in vivo*. Administration of SMe1EC2 protected endothelial function. It significantly decreased endothelaemia of diabetic rats and improved endothelium-dependent relaxation of arteries, slightly decreased ROS-production and increased bioavailability of ·NO in the aorta. Further studies with higher doses of SMe1EC2 may clarify the mechanism of its endothelium-protective effect *in vivo*.

## Introduction

In the last decades, scientific research has revealed that deterioration of the endothelium with subsequent damage of smooth muscle reactivity results in generalised increase in vascular tone, platelet aggregation, thrombus formation, etc. The endothelium plays an important role in maintaining vascular homeostasis by synthesising and releasing several vasodilators, including prostacyclin, nitric oxide radical (·NO), and endothelium-derived hyperpolarising factor (EDHF) (Vanhoutte and Mombouli, 1996; Shimokawa, 1999). Endothelial dysfunction participates in the pathophysiology of various diseases like atherosclerosis, essential hypertension, diseases connected with ischaemia/reperfusion (I/R), rheumatoid arthritis, diabetic micro- and macrovasculopathies, etc. Among the mechanisms of the vascular dysfunction observed in these diseases, increased generation of reactive oxygen species (ROS) is emerging as a crucial factor. This provides support for the use of antioxidants in preventing or alleviating the above mentioned diseases.

In the last decades, more than 70 new derivatives (Štolc *et al*., 2010) of the pyridoindole antioxidant stobadine – (–)-cis-2,8-dimethyl-2,3,4,4a,5,9b-hexahydro-1H-pyrido[4,3-b]indole, (Štolc *et al*., 1983; Horáková and Štolc, 1998) were synthesised and tested in the Institute of Experimental Pharmacology and Toxicology, Slovak Academy of Sciences. The main reason for synthesising new derivatives was to enlarge the antioxidative and antiradical capacity of the parent substance and to eliminate the undesired α_1_-adrenolytic activity of stobadine (Štolc *et al*., 2006). SMe1EC2, one of these new compounds, exhibited an increase in its antioxidative activity achieved by substitution of the methyl group in position 8 of stobadine by a methoxy group. This is explained by the increased ability of the aromatic circle to delocalise electron density from the stable nitrogen radical on indole. Substitution of nitrogen on the piperidine circle by an ethoxycarbonyl group prevented protonisation of this atom and led to loss of undesirable α_1_-adrenolytic activity and to decreased toxicity of SMe1EC2 (Štolc *et al*., 2010; Štolc *et al*., 2006; Májeková *et al*., 2006, Ujházy *et al*., 2008). This was the reason why we selected this compound to study the possibility to improve endothelial dysfunction in several experimental models.

In our previous works, we documented endothelial dysfunction induced by different pathological situations in experimental models of ischaemia/reperfusion (I/R) (Sotníková *et al*., 1998; Nosálová *et al*., 2004; Szöcs, 2004; Sotníková *et al*., 2005; Nosálová *et al*., 2007), adjuvant arthrithis (Sotníková *et al*., 2009), in spontaneously hypertensive rats in conditions of social stress (Sotníková *et al*., 2006, 2007), in hypertriglyceridaemic rats (Sotníková *et al*., 2008), etc. The common feature proved to be impaired endothelium-dependent relaxation of the arteries manifested by decreased response to acetylcholine. In the model of I/R of the abdominal aorta, we demonstrated I/R-induced reversible ultrastructural changes both in endothelial and smooth muscle cells, predominantly in the mitochondria. Participation of ROS in vascular injury was confirmed directly on using oxidative fluorescent microtopography (Szöcs *et al*., 2003), by observation of increased lipid peroxidation in homogenates of I/R aortae and by the ability of the antioxidant stobadine to protect the abdominal aorta against I/R-induced changes (Sotníková *et al*., 1998). Further, decreased release of NO_x_ and increased activity of lysosomal enzymes – N-acetyl-β-D-glucosaminidase (NAGA) and acid phosphatase (APH) – in aortic homogenates were observed (Sotníková *et al*., 1999). Lysosomes consist of a lipoprotein membrane surrounding potent acid hydrolases able to degrade all known constituents of cells. Leakage of lysosomal enzymes into cells and the surrounding extracellular space has been implicated in the pathogenesis of tissue injury (Waters *et al*., 1992). Pretreatment of rats with stobadine reduced the NAGA and APH release evoked by I/R (Sotníková *et al*., 1997, Navarová *et al*., 1998).

The use of the model of mesenteric I/R in rats provided evidence of ROS contribution to I/R-induced endothelial dysfunction. By the method of luminol enhanced chemiluminiscence (CL) we demonstrated increased generation of ROS in SMA tissue after 60-min ischaemia followed by 30-min reperfusion of the rat mesentery, which resulted in injury of both the ·NO and EDHF-endothelial relaxing systems of the SMA (Sotníková *et al*., 2005).

## Endothelial dysfunction in experimental diabetes

Although the aetiology of vascular disorders in long lasting diabetes is not fully understood, it has been suggested that abnormal vascular function may underlie many of the associated vasculopathies. These are major causes of disability and death in patients with diabetes mellitus (Yan *et al*., 2003). Under physiological conditions, glucose is transported into the cells by means of glucose transporters. Transport of glucose to the endothelium and smooth muscle cells is not insulin-dependent, thus in the case of hyperglycaemia intracellular concentration of glucose in these cells increases and can induce their injury.

Reduction in endothelium-dependent relaxation is a common feature known to occur in both conduit and resistance arteries of experimental diabetic animals. Moreover, increased responsiveness of vessels to contractile agents in diabetes mellitus was also reported. Among the proposed mechanisms of vascular dysfunction in diabetes, the increased generation of ROS is emerging as a crucial factor. The above facts provide support for the use of antioxidants in prevention of diabetic complications (Karasu *et al*., 1997, Afanasjev, 2010).

In our previous experiments, we found impaired endothelial function induced by long-lasting streptozotocin (STZ)-induced diabetes in rats (Sotníková *et al*., 1999a). Dietary supplementation of the pyridoindole antioxidant stobadine reduced this impairment (Sotníková *et al*., 2001). The long-lasting hyperglycaemia had a detrimental yet reversible effect on the structure of the aorta, which was reduced by administration of antioxidants (Okruhlicová *et al*., 2001).

The experimental model of STZ-induced diabetes was used to study the ability of the pyridoindole antioxidant SMe1EC2 to prevent or delay the onset of diabetic vasculopathies. Diabetes was induced by STZ (30 mg/kg i.p.) administered for three consecutive days to male rats. Administration of repeated low doses of STZ is connected with an autoimmune process, insulitis and slow death of pancreatic beta-cells, a process mimicking human diabetes type 1 (Lukic *et al*., 1991). Changes in vascular function (the aorta and SMA) and of markers of endothelial injury were studied after 5 and 10 weeks from the last dose of STZ.

**Figure 1:**
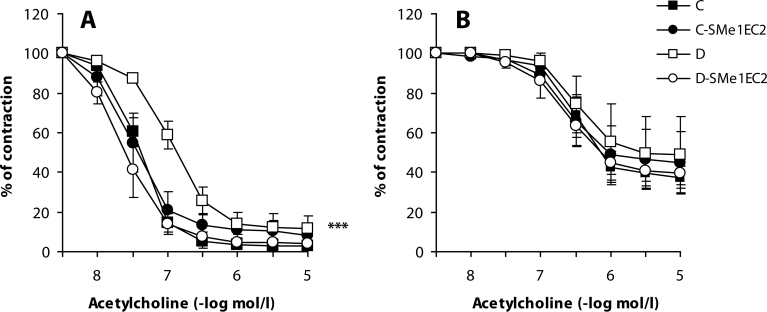
Effect of SMe1EC2 on endothelium-dependent relaxation of the superior mesenteric artery (SMA) *ex vivo*. Acetylcholine-induced responses of the phenylephrine (1µmol/l)-precontracted rings of SMA from control rats (C), control rats treated with 10mg/kg SMe1EC2 (C-SMe1EC2), diabetic rats (D), diabetic rats treated with SMe1EC2 (D-SMe1EC2). A – before blockade of eNOS with L-NAME and of prostaglandin synthesis with indomethacin; B – after blockade of eNOS with L-NAME and of prostaglandin synthesis with indomethacin. Data are means±S.E.M. of 8 experiments. ***p<0.001 *versus* C.

The first changes in vascular endothelial function could be observed 5 weeks after induction of diabetes and were demonstrated by increased endothelaemia (Table 1) and diminished endothelium-dependent relaxation of SMA to acetylcholine (Figure 1a). After prolonging the duration of diabetes to 10 weeks, the endothelial injury deepened and was observed also in the aorta (Zúrová *et al*. 2006).

**Table 1:** Effect of SMe1EC2 on endothelaemia, on basal chemiluminiscence and on NO_x_ and GSH levels in the aorta after 5 weeks of diabetes.

Group	Endothelaemia	CL	NO_x_	GSH
C	2.29 ± 0.29	0.573±0.13	108.82±11.21	4.34±0.70
D	3.71 ± 0.25*	1.153±0.18 *	61.90±14.42*	1.70±0.66*
D – SMe1EC2	2.05 ± 0.51^+^	0.925±0.12	89.85±5.02	2.78±0.62

C – control group, D – diabetic group, D-SMe1EC2 – diabetic rats treateted with SMe1EC2. Endothelaemia is expressed in the cell number/10µl blood, chemiluminiscence (CL) as AUC (area under the curve) in mV/min/mg aortic tissue, NOx levels in nmol/mg protein, levels of reduced glutathione (GSH) in µg/mg protein. Results are means ± S.E.M., n=5–9,* *p<*0.05 versus control. ^+^ *p<*0.05 versus diabetes

The reduced endothelium-dependent response can be mediated by several mechanisms: decreased bioavailability of ·NO, impaired diffusion of ·NO to smooth muscle cells, their decreased sensitivity or increased production of constrictory factors produced by the endothelium (Dai *et al*., 1993). In our experiments we demonstrated decreased ·NO concentration in the aorta after 5 weeks of diabetes (Table 1) accompanied by reduced response of SMA to acetylcholine. To find which endothelial factor was responsible for the reduction of acetylcholine-induced relaxation, we incubated arterial prepartions with the endothelial NO synthase (eNOS) inhibitor L-N^G^-nitroarginine methyl ester (L-NAME) and with the cyclooxygenase inhibitor indomethacin to obtain EDHF-dependent relaxation. In contrast to the results of Wigg *et al*. (2001), we did not find any changes in acetylcholine-induced relaxation after blockade of the ·NO-signalling pathway and prostaglandin synthesis (Figure 1b). This may be explained by the fact that those authors studied smaller branches of SMA where EDHF plays a more important role.

Diabetes-induced changes in ·NO-availability can be caused by changed production of ·NO. Adequate ·NO production depends on intact function of eNOS, with sufficiency of cofactors and substrates. Depressed production of ·NO can be the result of either decreased eNOS expression or its activity. The enzyme eNOS is localised in caveolae where it is coupled with caveolin-1, a22kDa protein, which is a physiological inhibitor of eNOS. After increasing intracellular calcium concentrations in endothelial cells, the complex caveolin-1 with eNOS breaks and eNOS is activated (Garcia-Cardena *et al*., 1997; Bucci *et al*., 2000). Using Western blot analysis, we did not find changes in eNOS expression, however expression of caveolin-1 was increased appr. to 140% of controls (*p<*0.05). We suggest that decreased ·NO bioavalability is not a consequence of reduced eNOS expression but of its activity caused by increased production of caveolin-1. These findings are in a good agreement with results of Bucci *et al*. (2004).

**Figure 2:**
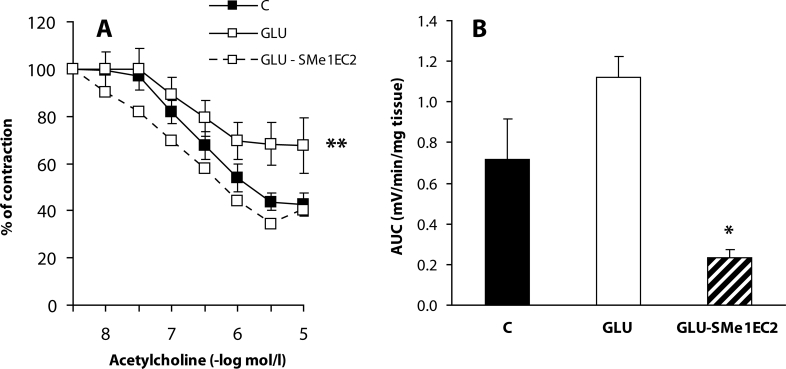
Effect of SMe1EC2 on endothelial dysfunction induced by a high concentration of glucose *in vitro.* C – control preparations incubated in Krebs solution, GLU – preparations incubated in Krebs solution containing 44 mmol/l glucose, GLU-SMe1EC2 – preparations incubated in Krebs solution containing 44 mmol/l glucose and 10 µmol/l SMe1EC2. A – responses of phenylephrine (1 µmol/l)-precontracted rings of the aorta to acetylcholine; B – chemiluminiscence expressed as AUC (area under the curve) in mV/min/mg of wet aortic tissue. Data are means±S.E.M. of 6 experiments. *p<0.05, **p<0.01 *versus* C.

Besides reduced ·NO production, increased inactivation of ·NO by ROS may participate in decreased ·NO bioavailabity. In conditions of oxidative stress, ·NO quickly reacts with superoxide creating peroxinitrite, which is cytotoxic for endothelial cells and oxidises tetrahydrobiopterin, an essential cofactor of eNOS. Consecutively, eNOS is uncoupled and preferentially produces superoxide instead of ·NO (Maritim *et al*., 2003). Moreover, peroxinitrite is also metabolised to a highly reactive hydroxyl radical (Beckman *et al*., 1990), which contributes to diabetes-induced endothelial dysfunction (Dai *et al*., 1993). Long-lasting heperglycaemia is the most important factor responsible for endothelial dysfunction in diabetes. Cosentino *et al*. (2003) observed reduced levels of NOx caused by incubation of endothelial cells in solutions containing high concentrations of glucose. Similarly in our *in vitro* experiments, incubation of the aorta in the solution containing high concentration of glucose resulted in reduced endothelium-dependent relaxation (Figure 2a). We suggest that this is connected with oxidative stress since on using CL we found increased ROS production in these aortic preparations (Figure 2b).

One of the sources of ROS production is activation of proteinkinase C (PKC), which activates NAD(P)H-oxidase producing high amounts of superoxide in vessels (Cosentino *et al*., 2003). NAD(P)H-oxidase is a dominant source of superoxide in the vessels. It is a multicomponent enzyme found in phagocytes, endothelial and smooth muscle cells and in fibroblasts of vascular adventitia. It consists of the main catalytic subunit gp91phox (in vessels nox2) and of regulatory subunists p22phox, p47phox, p67phox, p40phox and GTPase Rac2. The subunits gp91phox and p22phox supply the transport of electrons from NAD(P)H to O_2_. Homologues of gp91phox were idenitified in vessels and they are termed nox1-5. Up to 45–56% they are identical with gp91phox (Babior, 1999). In our experiments, we found increased ROS production already after 5 weeks of diabetes (Table 1) but no changes in mRNA expression of subunits nox1 and nox4. One of the explanations could be that in the early phases of diabetes enzymatic NAD(P)H-oxidase activity is increased by accelerated association of individual components without increased expression of catalytic subunits. However Wendt *et al*. (2005) observed increased activity simultaneously with increased nox1 expression after 8 weeks of diabetes. Another explanation could be that in the early phases of diabetes an increased production of superoxide in vessels may be the cosequence of increased expression of nox2 (Hink *et al*., 2001). It thus seems that individual catalytic subunits of NAD(P)H-oxidase are exprimed at different time intervals, in different types of cells and they may also possess different functions. While nox2 subunit is responsible for ROS production in endothelial cells, nox1 subunit is a source of ROS in vascular smooth muscle cells. Angiotensin II increases nox1 expression, yet it has no effet on nox4 subunit (Mollnau *et al*., 2002). On the other hand, TNF-α increases nox4 subunit and does not influence nox1 expression (Moe *et al*., 2006). In our experiments, we observed increased nox4 expression when diabetes lasted a very long time, *i.e.* 5 months (*p<*0.05), while nox1 expression was not changed. Moreover, nox4 expression was many times higher than nox1 expression. We suggest that under conditions of our experimental model, nox4 plays a more important role.

Oxidative stress can result also from reduced activity of antioxidative systems. We observed decreased levels of reduced glutathione (GSH) after 5 weeks of diabetes (from control values of 5.34±0.70 to 2.70±0.76 µg/mg protein, *p<*0.05) which is in agreement with results of Tachi *et al*. (2001) and Yue *et al*. (2003). Changes in GSH levels probably result from increased ROS production induced by hyperglycaemia, which is in accordance with the observation of GSH depletion in vascular cells incubated with a high concentration of glucose (Urata *et al*., 1996). On the other hand, after 10 weeks of diabetes GSH concentration in the aorta was not changed (Zúrová-Nedelčevová *et al*., 2006). We propose that unchanged GSH levels in the aorta may reflect adaptation of the organism to chronic oxidative stress in the later phase of diabetes.

## Effect of SMe1EC2

On using the described experimental model, an endothelium-protective effect of the antioxidant stobadine was observed. This finding confirmed the assumption of the important role of ROS in aetiopathology of diabetes-induced endothelial dysfunction (Sotníková *et al*., 2006a).

**Figure 3:**
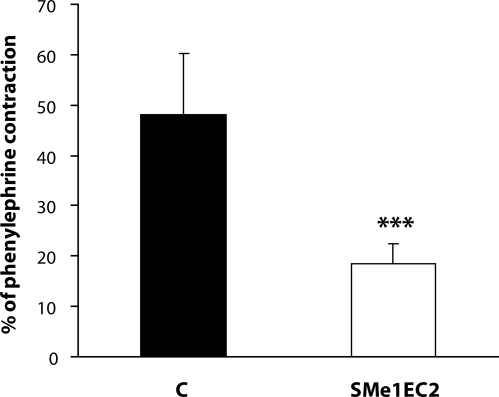
Effect of SMe1EC2 on the contraction induced by activated neutrophils *in vitro*. Responses of phenylephrine (1 µmol/l)-precontracted rings of the rat aorta to FMLP (N-formyl-methionyl-leucyl-phenylalanine, 10^−7^ mol/l)-activated neutrophils (10^6^/ml). C – control rings, SMe1EC2 – rings pretreated with 10µmol/l SMe1EC2. Data are means±S.E.M. of 12 experiments. ***p<0.001 *versus* C.

In the same model of STZ-diabetes, we further studied the effect of the stobadine derivative SMe1EC2 administered in the dose of 10 mg/kg every second day. A beneficial effect of SMe1EC2 on the vascular endothelium was found in our *in vitro* experiments, in which the aortal rings were incubated in a solution with a high concentration of glucose. The decreased endothelium-dependent relaxation was restored by this antioxidant (Figure 2). Searching for possible mechanisms of the SMe1EC2 protective effect, we found that it had no effect on basal tone of the arterial preparations or on contraction induced by depolarisation. It did not influence the concentration-response curves of phenylephrine or serotonin, indicating no effect on α_1_-adrenergic or 5-hydroxytryptamine receptors. Moreover, SMe1EC2 did not affect responses of the isolated aorta to the M_3_-agonist acetylcholine. Thus we propose that the endothelium-protective effect of SMe1EC2 is mediated by its antioxidant activity found also in the 1,1-diphenyl-2-picrylhydrazyl radical – (DPPH) test (Nosálová *et al*., 2010). With pD2 value of 5.67, SMe1EC2 exhibited a DPPH radical-scavenging activity higher than did stobadine. Indeed, under *in vitro* conditions, this compound suppressed production of ROS evoked by mesenteric I/R (Nosálová *et al*., 2010) and significantly reduced contraction of the aorta induced by ROS released from activated neutrophils (Bauer *et al*., 2008) (Figure 3).

We further found that *in vivo* administration of 10 mg/kg SMe1EC2 protected endothelial function. It significantly decreased endothelaemia of diabetic rats (Table 1) and improved endothelium-dependent relaxation of the aorta (Zúrová-Nedelčevová *et al*., 2006) and SMA without effect on the EDHF component (Figure 1). Therefore we assumed that this compound affected the ·NO-signalling pathway. Administration of SMe1EC2 slightly increased biological availability of ·NO in the aorta (Table 1), but it did not influence expression of eNOS or of caveolin-1. One of the explanations is that by its antioxidant properties SMe1EC2 inactivated ROS and thus increased ·NO bioavalability. This assumption was based also on the results of our *in vitro* experiments in which this compound intensively reduced CL increased by high glucose incubation and simultaneously protected endothelium-dependent relaxation of the aorta (Figure 2). In *in vivo* conditions, we observed a tendency of SMe1EC2 to decrease spontaneous ROS production and to increase levels of GSH in the aorta of diabetic rats (Table 1). These results may indicate that the dose of 10 mg/kg SMe1EC2 was not sufficient to observe all antioxidant properties *in vivo.* Further studies with higher doses of SMe1EC2 may clarify the mechanism of its endothelium-protective effect in STZ-diabetes in rats.
